# P300 as a correlate of false beliefs and false statements

**DOI:** 10.1002/brb3.3021

**Published:** 2023-04-19

**Authors:** Yang Wang, Carrey Tik Sze Siu, Him Cheung

**Affiliations:** ^1^ Department of Behavioural and Social Sciences The City University of Hong Kong Hong Kong SAR China; ^2^ Department of Early Childhood Education The Education University of Hong Kong Hong Kong SAR China; ^3^ Department of Psychology The Education University of Hong Kong Hong Kong SAR China

**Keywords:** false belief, lie, LSW, P300, theory of mind

## Abstract

**Introduction:**

This study investigates P300 as a component for false belief and false statement processing with and without a communicative context. The purpose is to understand why P300 has been shown to be commonly involved in false belief and lie processing.

**Methods:**

Participants were presented with a story in which the protagonist holds a true belief and makes a true statement of it (true belief), holds a false belief and makes a true statement (false belief), or holds a true belief and makes a false statement (false statement) while electroencephalograms were recorded.

**Results:**

In Experiment 1, featuring a solitary protagonist, stronger posterior P300 was shown in the false belief condition than the true belief and false statement condition. With the installation of a communicative context by including a second character listening to the protagonist, Experiment 2 showed enhanced frontal P300 in the false statement condition compared to the true belief and false belief condition. A late slow wave was more prominent in the false belief condition than in the other two conditions in Experiment 2.

**Conclusion:**

The present results suggest a situation‐dependent nature of P300. The signal captures the discrepancy between belief and reality more readily than that between belief and words under a noncommunicative context. It becomes more sensitive to the discrepancy between belief and words than that between belief and reality in a communicative situation with an audience, which makes any false statement practically a lie.

## Graphical abstract

P300 is sensitive to belief‐reality and belief‐words discrepancies in noncommunicative and communicative situations, respectively. This explains why the component is commonly involved in false belief and lie understanding.

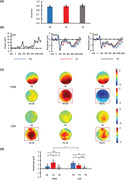

## 1 INTRODUCTION

In many life circumstances, we are interested in knowing what others believe and comparing what they say they believe to our own understanding, because such information guides our interaction with them and supplements our cognition about the world. An agent is said to possess a false belief if what she believes deviates from reality, and to make a false statement if her words deviate from her belief. A false statement is considered a lie if it is directed towards an audience in a communicative context. In the present study, we examine the role of P300 in representing discrepancies between belief and reality on the one hand and between belief and words on the other. We are concerned with situations in which an agent makes a true (honest) or false (dishonest) statement about her belief that agrees with (true belief) or contradicts reality (false belief). Is P300 differentially sensitive to false beliefs and false statements in a communicative versus a noncommunicative context, only the former of which may turn a false statement into a lie?

False belief understanding requires the representation of an alternative belief that deviates from the observer's own understanding of reality (Wellman & Peterson, 2013). Previous event‐related potential (ERP) studies have shown that false belief is associated with P300 and a late slow wave (LSW). P300 typically shows a parietal distribution and increased positivity for belief compared to reality reasoning, and for false compared to true belief reasoning (Meinhardt et al., 2011; Sabbagh & Taylor, 2000). Large P300 amplitudes for false belief are associated with activities in the temporo‐parietal junction (TPJ) (Cheung et al., 2012; Meinhardt et al., 2011), a region that is implicated in belief thinking and especially false belief reasoning (Sommer et al., 2007). The involvement of the TPJ in false belief reasoning has been taken to indicate the working of the attention system in detecting salient and task‐related stimuli in the test environment (Corbetta et al., 2008). Because false belief features discrepancy between an agent's epistemological state and reality, the deviation may draw the observer's attention from reality to the agent's mental world. In a study investigating ERP responses to false beliefs, Meinhardt et al. (2011) observed that a late positive complex in the 300–600 ms time window with a posterior distribution might be a “P3‐like component” associated with reorientation from external stimuli to internal mental representations. This interpretation is compatible with the general characteristics of P300 with a temporal‐parietal distribution, that is, P3b, suggesting an attentional shift from reality to the agent's mental model of reality, which needs to be updated (Kirino et al., 2000; Polich, 2007).

In addition to P300, LSW is also considered an ERP component associated with false belief. Differences in LSW between belief and reality processing have been observed with frontal and parietal distributions (Chen et al., 2012; Geangu et al., 2013; Liu et al., 2009, 2004; Meinhardt et al., 2012; Sabbagh & Taylor, 2000). For instance, Liu et al. (2009) observed an LSW with a frontal distribution that was associated with desire and belief judgment, and an LSW with a posterior distribution that was only associated with belief judgment. The frontal LSW may indicate the working of a general neural system for mental state reasoning, while the posterior LSW may have to do with specific neural processes behind belief reasoning (Bowman et al., 2012). Zhang et al. (2013) observed that LSW for false belief was more positive at frontal, central, and parietal sites than that for false sign, suggesting that LSW might reflect processes specific to belief reasoning. Zhang et al. (2013) also reported that LSW was more positive for false than true belief thinking.

While false belief involves a discrepancy between the false content of an epistemological state and reality, a false statement is made when an epistemological state is misrepresented by a verbal description of it. A false statement becomes a lie when it is directed to an audience with an assumed deceptive intention (Cheung et al., 2015; Chisholm & Feehan, 1977; Peterson, 1995; Sullivan et al., 1995; Wimmer & Perner, 1983). Interestingly, previous studies have reported a behavioral correlation between false belief and lie telling (Baron‐Cohen, 1992; Talwar & Lee, 2008) and lie understanding (Cheung et al., 2015; Hsu & Cheung, 2013; Maas, 2008), which may be partly explained by the general association between theory of mind and language use (e.g., Cheung, 2006; Cheung et al., 2004, 2009; Siu & Cheung, 2022). Past ERP studies have also shown that P300 is an important component in lying as in false belief. In their meta‐analysis, Leue and Beauducel (2019) showed that the amplitude of P3b, a P300 with a more posterior distribution, could be used to distinguish between concealed and truthful knowledge. Gibbons et al. (2018) found both P3b and P3a (a relatively frontal P300) were associated with the detection of deception. Mei et al. (2020) observed larger P300 amplitudes elicited by lies than the truth told by outgroup members. Such P300 differences may reflect differences in implicit attitude and allocation of cognitive and neural resources between perception of lies and truthful statements, especially when the lie is interpreted as an arousing stimulus indicating potential danger from outgroup individuals. This is consistent with previous findings associating P300 with the amygdala (Halgren et al., 1980), which plays a key role in emotion processing (Davis & Whalen, 2001).

To summarize, previous ERP research suggests that false belief could be delineated via the examination of P300 and LSW while the telling and perception of lies correlate primarily with P300. The present study investigates why P300 is commonly involved in the two domains. We consider the possibility that P300 indicates the decoupling of representations that is highlighted by the situation. A noncommunicative false belief situation highlights the discrepancy between belief and reality, alerting the observer to the mental representation of the agent that needs to be updated (Meinhardt et al., 2011). The departure of the agent's words from her belief should figure less prominently in a noncommunicative context because it is not at all obvious to whom she is directing her words. Hence, P300 in this situation is assumed to indicate primarily the decoupling of the agent's false belief from reality. In a lie situation, which is by nature communicative, on the other hand, whether the liar's belief is consistent with reality is less important. What defines a lie is the departure of the liar's words from her genuine belief, which may be true or false, in a context highlighting the communicative relationship between the liar and the lie recipient. In this situation, P300 is assumed to indicate primarily the decoupling of the liar's words from her belief. P300 in this communicative situation may be less sensitive to any belief‐reality discrepancy. In short, we hypothesize that P300 is differentially sensitive to false belief and false statement depending on whether communication is highlighted by the situation.

To test this situated view of P300 as an index of decoupling, the present study examines the component in three conditions featuring, respectively, a true belief, a false belief, and a false statement. We use a picture‐statement paradigm modeled after Meinhardt et al. (2012) to present test scenarios, in which the story protagonist claims that a certain animal *is in a cage* while actually seeing it there (true belief with a true statement, or TB), wrongly assuming that it is still there (false belief with a true statement, or FB), or seeing it run away (true belief with a false statement, or FS). The story in Experiment 1 does not include an audience; the discrepancy between the statement and the belief in FS figures less prominently because the words do not serve any apparent purpose. The departure of the protagonist's false belief from reality in FB is the more important discrepancy highlighted by the situation. Therefore, enhanced P300 in the FB condition is compared to the other two conditions. P300 may also be enhanced in FS compared to TB because there is a statement‐belief discrepancy in FS, albeit a less important discrepancy because it is not clear to whom the statement is directed. In Experiment 2, there is always a second character listening to the protagonist, and thus, the false statement in FS becomes a lie. In this communicative situation, the discrepancy between the statement and the belief is highlighted more than that between reality and the belief. Therefore, enhanced P300 in FS is expected compared to the other two conditions. FB may differ from TB in P300 because of a belief‐reality discrepancy, but to a smaller extent compared to Experiment 1.

## 2 EXPERIMENT 1

### 2.1 Materials and methods

#### 2.1.1 Participants

Twenty Chinese‐speaking undergraduates at the Chinese University of Hong Kong participated in this study after giving informed consent. The number of participants was determined with a power analysis using the G*Power software (Erdfelder et al., 1996), indicating that a sample of 18 participants was needed to detect differences with a repeated measures ANOVA with power (1 − *β*) set at 0.80, alpha at 0.05, and an estimated *ŋ_p_^2^* of 0.06, a medium effect size by Cohen's (1988) standard. The selection of a medium effect size was based on its use in previous ERP studies on false belief processing (e.g., Mei et al., 2023; Wang et al., 2021). They were right‐handed, had normal hearing, and normal or corrected‐to‐normal vision with no history of psychiatric or neurological disorders. Four participants were excluded because of low response accuracy (<65%) (3) and excessive electroencephalogram (EEG) artifacts (1). Sixteen participants were included in the final sample (13 females, mean age = 19.8 years; *SD* = 1.7 years). The study was approved by the Survey and Behavioral Research Ethics Committee, Social Sciences Panel of the Chinese University of Hong Kong.

#### 2.1.2 Materials and procedure

**FIGURE 1:**
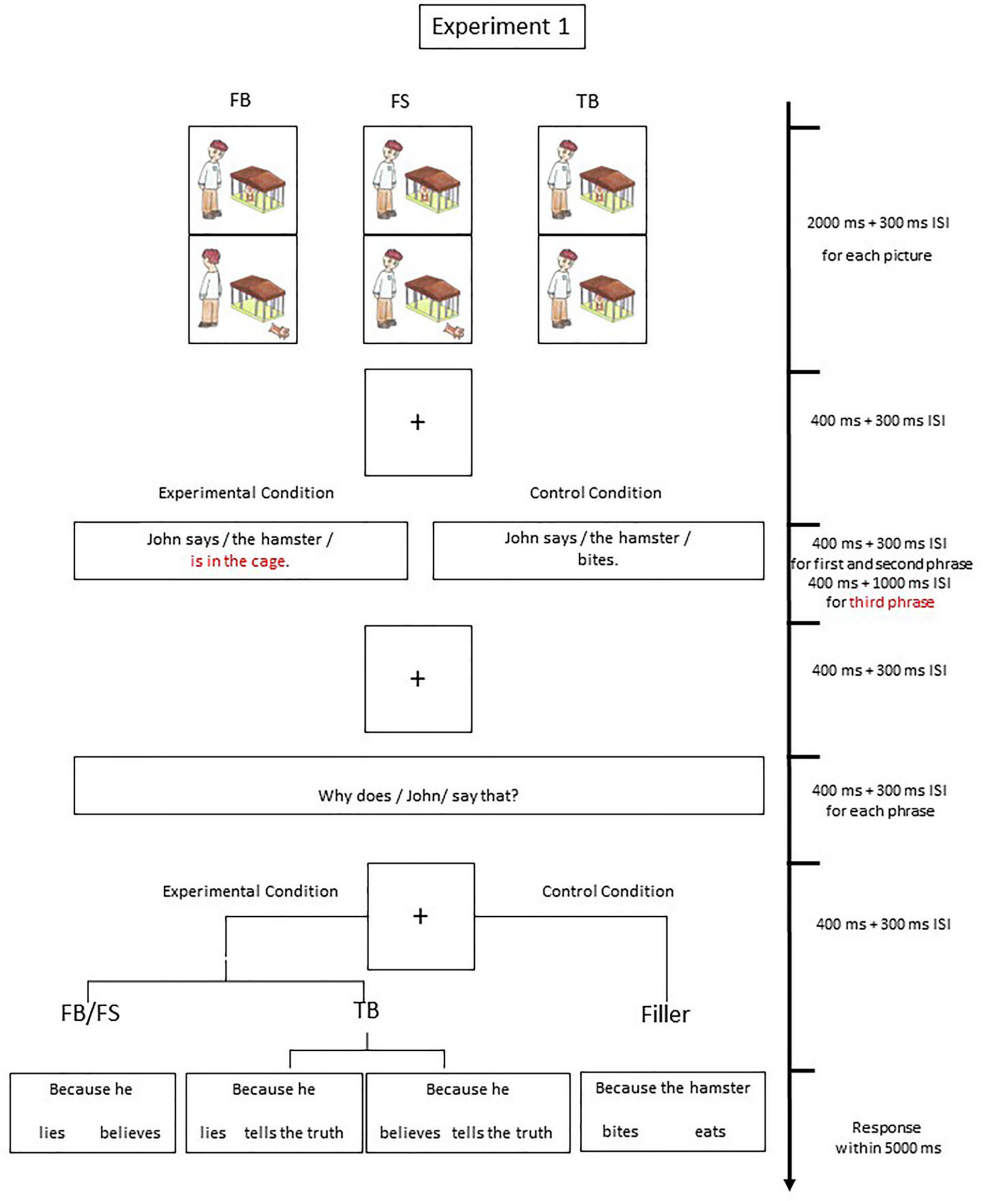
Example of an FB, FS, and TB story: In all three conditions, the phrase “*is in the cage*” was the ERP eliciting event. To prevent anticipation, control (filler) trials were randomly interspersed between the experimental trials. Control trials were not included in the ERP analysis.

The task was modified by Meinhardt et al. (2012), who explored the similarities and differences in ERP responses between false belief reasoning and pretense. Three within‐subject experimental conditions were devised: False Belief (FB), False Statement (FS), and True Belief (TB). The trial structures in the three conditions were identical (see Figure 1). In the first slide, the protagonist sees an animal (e.g., hamster) sitting in a cage. The second slide differed across the experimental conditions. In FB, the protagonist turns back and cannot see the animal escape from the cage; in FS, he or she witnesses the animal's escape. In TB, the protagonist sees the animal still sitting in the cage. A claim made by the protagonist was then presented, “John says a hamster is in the cage.” The phrase “in the cage” was the critical ERP event because false belief, false statement, and truth telling were distinguished at this point given the different situations depicted in the second slide. Next, the test question was presented, “Why does John say that?” Participants chose the answer from two options, “because he lies” versus “because he believes” in FB and FS, and “because he lies” (or “because he believes”) versus “because he tells the truth” in TB.

Fillers having well‐matched contents and story structures were presented as control trials, in which the protagonist's claim was about things other than the location of the animal, for example, “The hamster bites.” The gender of the protagonist (two female and two male characters), color of the cage (brown, blue, yellow), and the animal (hamster, squirrel, dog, rabbit, mouse, duck, and cat) were counterbalanced across conditions. Each participant was subjected to all the conditions in a within‐subject design. All verbal material was presented in written Chinese.

Participants sat in a comfortable chair in a sound‐attenuated, dimly lit room and were placed with an elastic cap (Electro‐Cap International). They first went through a 12‐trial practice session with corrective feedback and then the experimental session, comprising six blocks of 42 trials. A trial started with two picture slides, each presented for 2000 ms followed by a 300‐ms interstimulus interval (ISI, blank screen). A 400‐ms fixation cross then appeared, followed by a 300‐ms ISI, and the protagonist's claim was broken into three short phrases presented in three respective slides to follow, each lasting 400 ms and followed by a 300‐ms ISI except for the last, that is, the critical ERP event, which was followed by a 1000‐ms ISI. A fixation cross appeared for 400 ms, followed by a 300‐ms ISI and then three slides, each containing a component phrase to the test question, separated by 300‐ms ISIs. A fixation cross of 400 ms was then presented, followed by a 300‐ms ISI and then the answer options. The participant would respond in 5000 ms by pressing one of two designated keys. Response accuracies and response times were recorded. In each condition, participants completed 42 experimental and 42 control trials, which were all randomly presented. Blocks of trials were separated by 2‐minute breaks.

Stimulus presentation was controlled by E‐prime (Schneider et al., 2002) and data were analyzed with the EEGLAB Toolbox (version 13; Delorme & Makeig, 2004) of the MATLAB software (version 2012a; MathWorks, Natick, MA).

#### 2.1.3 Electrophysiological recordings

The EEG was acquired with 64 Ag/AgCl electrodes placed according to the International 10–20 system using the NeuroScanSynAmps system (Charlotte, NC, USA). All electrodes were referenced to the left mastoid in on‐line recording and re‐referenced to the average of the left and right mastoids in off‐line analyses. The forehead (Afz) was used as the ground electrode. Electrooculogram (EOG) signals were obtained from above versus below the left eye (vertical EOG) and the left versus right lateral orbital rim (horizontal EOG) to monitor vertical eye movements or blinks and horizontal eye movements. Electrode impedances were maintained below 5 kΩ. Signals were continuously sampled to the hard disk at a digitization rate of 500 Hz.

#### 2.1.4 Behavioral data analysis

The analysis of behavioral data examined whether the participants showed different accuracies among the three experimental conditions in answering the test question, “Why does John say that?” A one‐way ANOVA was conducted on the accuracy data with condition (FB, FS, TB) as the independent factor.

#### 2.1.5 EEG data analysis

Offline analyses were conducted in Neuroscan Scan 4.5 (Neuroscan, Inc.). EEG signals were rereferenced to the average of the left and right mastoids. The digitized EEG data were rereferenced to the average of both mastoid electrodes, and the bandpass was filtered at 0.1−30 Hz. The filtered data were segmented into epochs of 1400 ms, including a prestimulus baseline of 200 ms, time‐locked to the onset of the critical phrase, “in the cage”. Ocular artifacts were corrected by using a classical regression method (Gratton et al., 1983). Only epochs with an amplitude change smaller than 150 μV were retained for further analyses. ERP waveforms were generated by averaging epochs for each condition, channel, and participant. The analyses included only the experimental trials in which correct responses were recorded. On average, 28, 27, and 27 trials were included in the final analysis for FB, FS, and TB, respectively. The numbers of trials analyzed did not differ among the three conditions, *F*(2, 30) = 0.552, *p* = .582, *ŋ^2^_p_* = .035.

#### 2.1.6 Global field power analysis

**FIGURE 2:**
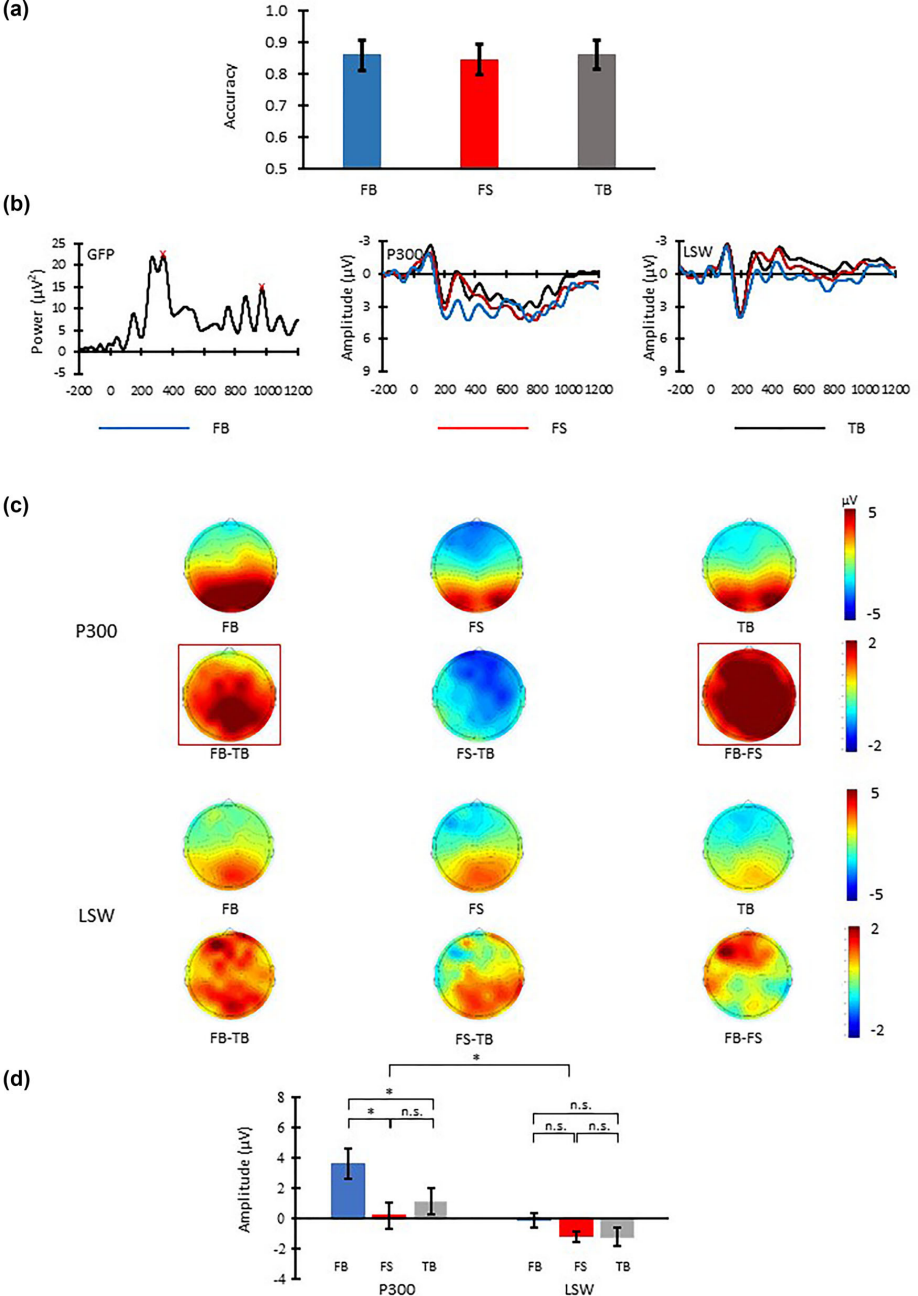
Behavioral and ERP results of Experiment 1. (a) Accuracies in the three conditions. (b) Left panel: Global field power of mean sum of squares peaking (red cross) at 340 and 970 ms in the two target time windows (i.e., 250–450 and 700–1200 ms); middle panel (P300): Grand average ERP waveforms across CPz and its surrounding electrodes (i.e., C1, Cz, CP1, CP2, P1, Pz, and P2); right panel (LSW): Grand average ERP waveforms across F3 and its surrounding electrodes (i.e., FP1, AF3, F5, F1, FC5, FC3, and FC1). (c) Scalp distributions of P300 (330–350 ms) and LSW (870–1070 ms) in the three conditions and between‐condition differences. (d) Mean P300 and LSW amplitudes. Error bars indicate SEM computed across participants; *p < .05.

In the Global Field Power (GFP) analysis, we focused on P300 (250–450 ms) and LSW (700–1200 ms), following previous ERP studies investigating false belief understanding (Meinhardt et al., 2012, 2011). In each condition, GFP waveform amplitudes were produced by averaging the mean sums of squares from all the electrodes at different time points (left panel, Figure 2b). The GFP waveforms represented variances in the spatial distribution of brain responses across time and were used to identify the time windows and electrodes showing differences among the three conditions. Peaks of GFP corresponding to P300 (340 ms) and LSW (970 ms) were identified in the 250–450 and 700–1200 ms search window, respectively. A 20‐ms measurement time window centering on the P300 peak (i.e., 330–350 ms) and 200‐ms measurement time window centering on the LSW peak (i.e., 870–1070 ms) were used to calculate the P300 and LSW responses. The CPz and F3 electrodes were identified with the strongest response in the P300 and LSW measurement time window, respectively.

In each condition, P300 and LSW from CPz and F3 and their neighboring electrodes were averaged and submitted to a repeated‐measures ANOVA with the independent factors: Electrodes and Interval of Interest (EIOI; averaged P300 responses at CPz, C1, Cz, CP1, CP2, P1, Pz, P2 within the 330–350 ms range, averaged LSW responses at F3, FP1, AF3, F5, F1, FC5, FC3, FC1 within the 870–1070 ms range), and condition (FB, FS, TB). Follow‐up *t*‐tests were performed using the Bonferroni method to adjust the critical *p* or alpha value for type l error.

#### 2.1.7 Randomization tests

In the GFP analysis, the selection of time windows and electrodes was based on peak measurements, which could be sensitive to random noise. To evaluate possible measurement bias, nonparametric randomization tests equivalent to the mentioned repeated‐measures ANOVA, follow‐up one‐way ANOVA, and *t*‐tests were conducted in addition to the main analysis. The results of these nonparametric randomization tests (see Supporting Information Analysis and Results) were similar to the results of the main analysis reported below.

All the data reported, test materials, and analysis code used in this experiment are available from the first author. This study was not preregistered.

### 2.2 Results and discussion

#### 2.2.1 Behavioral results

There was not an overall difference in response accuracy among FB, FS, and TB, *F*(2, 30) = 0.789, *p* = .463, *ŋ^2^_p_* = .05. See Figure 2a.

#### 2.2.2 ERP results

Figure 2b (middle and right panel) and 2C, respectively show the averaged ERP waveforms in the three conditions and the scalp distribution of between‐condition differences in P300 and LSW. Repeated‐measures ANOVA showed significant main effects of EIOI, *F*(1, 15) = 10.067, *p* = .006, *ŋ^2^_p_* = 0.402, and condition, *F*(2, 30) = 5.325, *p* = .01, *ŋ^2^_p_* = .262. The interaction was also significant, *F*(2, 30) = 4.375, *p* = .035, *ŋ^2^_p_* = .226). Follow‐up one‐way ANOVA with condition as independent factor was conducted on P300 and LSW separately. P300 differed among the three conditions, *F*(2, 30) = 6.395, *p* = .013, *ŋ^2^_p_* = .299, whereas LSW did not, *F*(2, 30) = 1.896, *p* = .168, *ŋ^2^_p_* = .112. Post‐hoc analyses revealed that FB elicited larger P300 than both TB, *t*(15) = 2.194, *p* = .044, and FS, *t*(15) = 2.989, *p* = .009. FS and TB did not differ in P300, *t*(15) = −1.597, *p* = .131. No differences among the three conditions were found for LSW (*t*s = 0.072 to 1.543, *p*s = .090 to .144).

#### 2.2.3 Discussion

As expected, FB elicited stronger P300 than FS and TB, indicating decoupling of the protagonist's false belief from reality. A reality‐belief conflict was present only in FB; the protagonist's belief was always consistent with reality in FS and TB. Previous studies have suggested an association between P300 and TPJ during false belief reasoning (Meinhardt et al., 2011) while TPJ activities are an important component to the overall theory of mind network (Aichhorn et al., 2009). Note that a conflict between the story protagonist's and the participant's belief has been central to many widely used ToM paradigms in which decoupling of conflicting interpretations of reality is highlighted as a proxy for ToM. The present observation that FB was associated with stronger P300 than FS, in which the false statement is not received by an audience in the story, provides support for the view that P300 is more sensitive to the representational discrepancy highlighted than not highlighted by the situation. The false statement in FS does not serve a communicative purpose, because it is not obvious to whom the statement is directed, and hence, its discrepancy from belief is considered less important by the observer in this situation.

Unexpectedly, the present results did not show a difference in LSW between FB and TB or FS. This is inconsistent with previous studies showing parietal LSW differences between belief and photo representations (Sabbagh & Taylor, 2000), false belief and true belief (Geangu et al., 2013; Zhang et al., 2013), and false belief and reality (Kühn‐Popp et al., 2013). The LSW has been shown to be especially responsive to processing of belief states compared with other mental states such as desire (Bowman et al., 2012; Liu, Meltzoff et al., 2009). The present lack of a condition effect on LSW awaits further examination.

## 3 EXPERIMENT 2

Experiment 1 showed that P300 with a posterior distribution was enhanced in a false belief situation without a communicative context, in which discrepancy between the protagonist's false thought and reality was highlighted. Deviation of the false statement from reality and the true belief in FS did not produce a similar effect. In Experiment 2, a communicative context was installed by including an audience into the story listening to the protagonist, turning the false statement into a lie. P300 in the FS condition was expected to be enhanced relative to the other two conditions, which did not contain a false statement, if the signal was sensitive to the representational discrepancy featured by the situation, assuming that the communicative context highlighted the importance of the discrepancy between belief and words, not that between belief and reality. P300 might also be enhanced in FB because of a belief‐reality discrepancy, compared to TB. Yet the magnitude of this effect should be much smaller than that in Experiment 1 because belief‐reality discrepancy was not highlighted by the communicative context in the current experiment.

### 3.1 Materials and Methods

#### 3.1.1 Participants

Twenty Chinese‐speaking undergraduates at the Chinese University of Hong Kong participated after giving informed consent. The number of participants was determined in accordance with a power analysis using the G*Power software (Erdfelder et al., 1996), which indicated that a sample of 18 participants was needed to detect differences with a repeated measures ANOVA with power (1 − *β*) set at 0.80, alpha at 0.05, and an estimated *ŋ_p_^2^* of 0.06 (medium) (Cohen, 1988; Mei et al., 2023; Wang et al., 2021). They were all right‐handed, had normal or corrected‐to‐normal vision and normal hearing, and were not diagnosed with any psychiatric or neurological disorders. Two participants were excluded because of their low response accuracy (<65%). Eighteen participants were included in the final sample (10 females, mean age = 20.7 years; *SD* = 1.9 years). This study was approved by the Survey and Behavioral Research Ethics Committee, Social Sciences Panel of the Chinese University of Hong Kong.

#### 3.1.2 Materials and procedure

**FIGURE 3:**
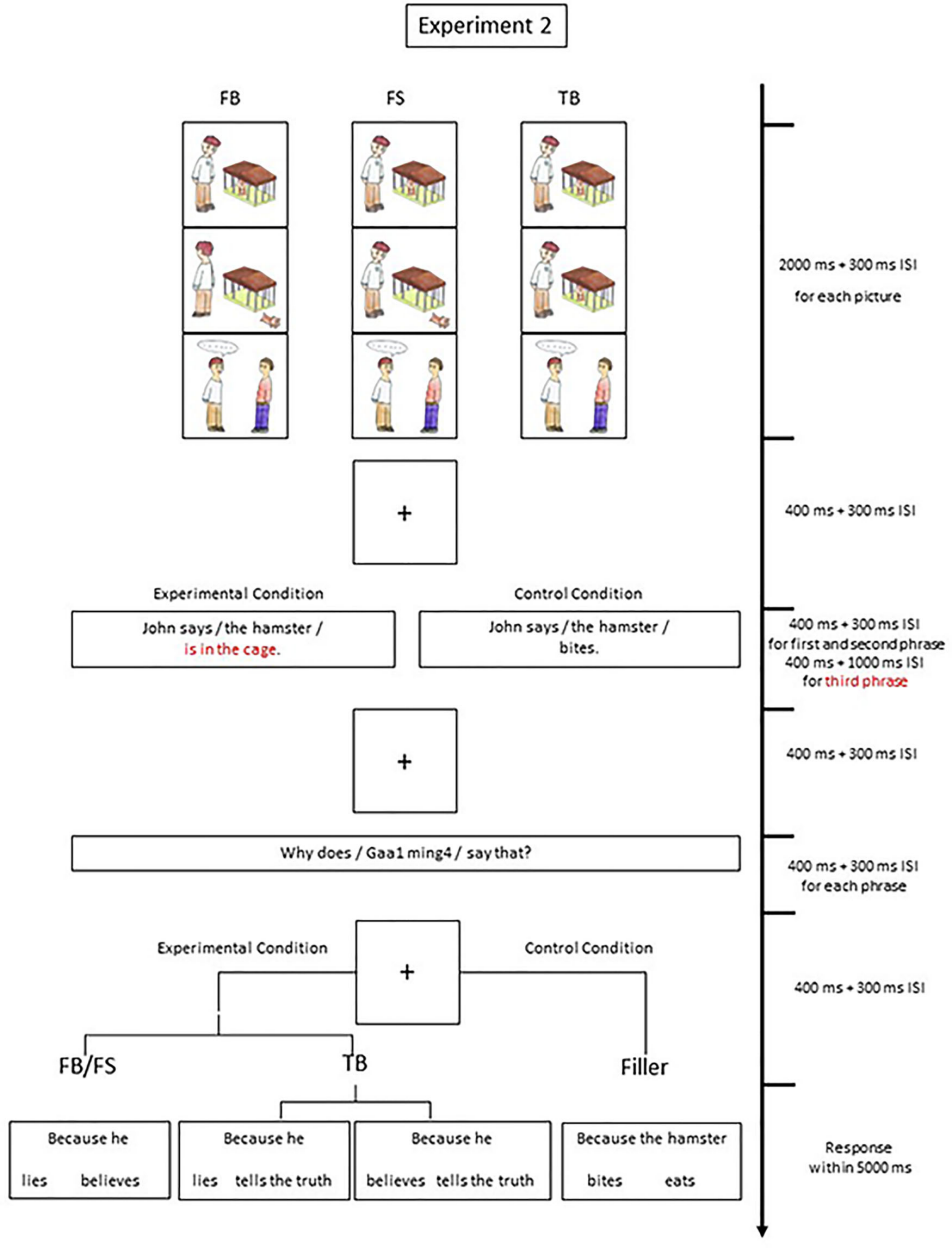
Example of an FB, FS, and TB story: In all three conditions, the phrase “*is in the cage*” was the ERP eliciting event. To prevent anticipation, control (filler) trials were randomly interspersed among the experimental trials. Control trials were not included in the ERP analysis.

The materials and procedure were the same as in Experiment 1, except that a third picture slide was added showing the protagonist talking to another character after his/her encounter with the animal, prior to the presentation of the word slides. This second character listens to the protagonist in different conditions. See Figure 3.

#### 3.1.3 Electrophysiological recordings, behavioral data analysis, EEG data analysis, GFP analysis, and randomization tests

**FIGURE 4:**
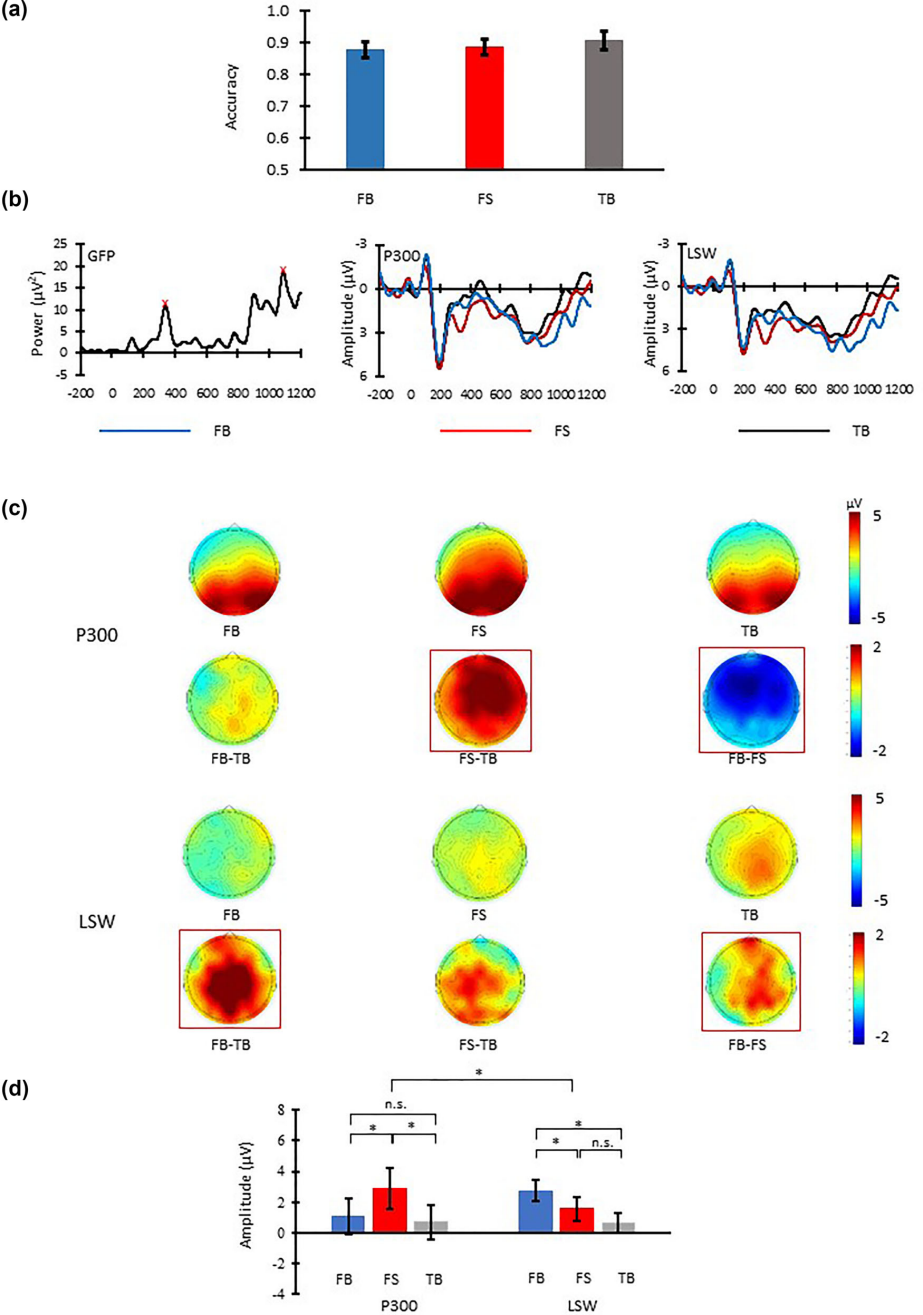
Behavioral and ERP results of Experiment 2. (a) Accuracy in the three conditions. (b) Left panel: Global field power of mean sum of squares peaking (red cross) at 338 and 1088 ms in the two target time windows (i.e., 250–450 and 700–1200 ms); middle panel (P300): Grand average ERP waveforms across FCz and its surrounding electrodes (i.e., F1,Fz, F2, FC1, FC2, Fz, and C2); right panel (LSW): Grand average ERP waveforms across C2 and its surrounding electrodes (i.e., FCz, FC2, FC4, Cz, C4, CPz, CP2, and CP4). (c) Scalp distributions of the P300 (328–348 ms) and LSW (988–1188 ms) in the three conditions and between‐condition differences. (d) Mean P300 and LSW amplitudes. Error bars indicate SEM computed across participants; *p < .05.

Experiment 2 adopted the same recording and analysis protocol as Experiment 1. On average, 31, 30, and 31 trials were included in the final analysis for FB, FS, and TB, respectively. The numbers of trials analyzed did not differ among the three conditions, *F*(2, 34) = 0.891, *p* = .420, *ŋ^2^_p_* = 0.050. Peaks of the GFP corresponding to P300 (i.e., 338 ms) and LSW (i.e., 1088 ms) responses were identified in the 250–450 and 700–1200 ms search windows, respectively (left panel, Figure 4b). A 20‐ms measurement time window centered on the P300 peak (i.e., 328–348 ms) and 200‐ms measurement time window centered on the LSW peak (i.e., 988–1188 ms) were used to calculate the P300 and LSW responses. Electrodes (i.e., FCz and C2) with the strongest responses in the P300 and LSW measurement time windows were identified.

The P300 and LSW in the three conditions at these electrodes and their corresponding neighboring electrodes were averaged and submitted to a repeated‐measures ANOVA with the independent factors (EIOI; averaged P300 response at FCz, F1, Fz, F2, FC1, FC2, Fz, C2 within 328–348 ms, averaged LSW response at C2, FCz, FC2, FC4, Cz, C4, CPz, CP2, CP4 within 988–1188 ms) and condition (FB, FS, TB). For follow‐up *t*‐tests, the Bonferroni method was adopted to adjust the critical *p*‐value or alpha‐value for multiple comparison correction. Nonparametric randomization tests equivalent to the mentioned repeated‐measures ANOVA, follow‐up one‐way ANOVA, and *t*‐tests were conducted. The results of the nonparametric randomization tests were similar to the results of the main analysis reported below (see Supporting Information Analysis and Results).

All the data reported, test materials, and analysis code used in this experiment are available from the first author upon reasonable request. This study was not preregistered.

### 3.2 Results and discussion

#### 3.2.1 Behavioral results

There was not an overall difference among the conditions (FB, FS, TB) in response accuracy, *F*(2, 34) = 1.33, *p* = .28, *ŋ^2^_p_* = .07. See Figure 4a.

#### 3.2.2 ERP results

Figures 4b (middle and right panels) and 4C show the averaged ERP waveforms in the three conditions and the scalp distribution of between‐condition differences in P300 and LSW. The main effect of condition was significant (*F*[1, 17] = 4.514, *p* = .018, *ŋ^2^_p_* = .210), whereas the main effect of EIOI was not (*F*[1, 17] = 0.015, *p* = .905, *ŋ^2^_p_* = .001). The two‐way interaction was significant, *F*(2, 30) = 7.616, *p* = .002, *ŋ^2^_p_* = .309). Follow‐up one‐way ANOVA with condition as independent factor was conducted separately on P300, *F*(2, 34) = 4.732, *p* = .015, *ŋ^2^_p_* = .218, and LSW, *F*(2, 34) = 7.041, *p* = .003, *ŋ^2^_p_* = .293. Significant between‐condition differences were found. Post‐hoc analyses revealed that FS elicited larger P300 than both FB, *t*(17) = 2.151, *p* = .046, and TB, *t*(17) = 2.657, *p* = .017. FB and TB did not differ in P300, *t*(17) = 0.631, *p* = .537, but FB did elicit a larger LSW than both FS, *t*(17) = 2.360, *p* = .030, and TB, *t*(17) = 3.623, *p* = .002. FS and TB did not differ in LSW, *t*(17) = 1.525, *p* = .146.

#### 3.2.3 Discussion

With the addition of a third picture slide showing the story protagonist's talking to a second character, Experiment 2 showed stronger P300 with a frontal distribution in the FS than the FB and TB conditions. This is consistent with the notion that a communicative context highlights the discrepancy between an agent's belief and words, which is picked up by an enhanced P300 response. Together with the findings of Experiment 1, the present results suggest that P300 captures the representational discrepancy that is highlighted by the situation: Whether the agent holds an accurate belief about reality becomes less important than whether she is honest when communicating her belief to another individual. The lack of a significant P300 difference between FB and TB is consistent with the assumed unimportance of belief‐reality discrepancy in the present communicative situation. The current results also help explain why P300 has been shown to be commonly involved in false belief and lie understanding in the literature. In a typical false belief situation which is noncommunicative, enhanced P300 probably reflects the departure of the false belief from reality and an attention shift toward the mental world of the agent (Meinhardt et al., 2011). In a lie situation (i.e., FS in Experiment 2), the basis of the enhanced P300 may be the discrepancy between the liar's words and her belief. Relatively little attention is paid to the veracity of the belief.

Unlike Experiment 1, a more pronounced LSW was shown in FB than in FS and TB, while no LSW difference was demonstrated between FS and TB. This result is consistent with the literature reporting LSW as a correlate of false belief understanding (e.g., Meinhardt et al., 2012).

## 4 GENERAL DISCUSSION

In the present study, Experiment 1 showed enhanced posterior P300 in FB compared to FS and TB without a second character listening to the protagonist in the story. FS and TB did not differ. In contrast, Experiment 2 showed enhanced P300 with a more frontal distribution in FS compared to FB and TB, with a second character listening to the story protagonist. FB and TB did not differ. We argue that the presence of the second character in Experiment 2 changes the nature of the situation as perceived by the participants, directing their attention from the discrepancy between belief and reality (FB) to that between belief and words (FS) because the words are now listened. The false statement in Experiment 2 has become a lie. The present findings help explain why P300 has been shown to be commonly involved in false belief and lie understanding: It is because both domains feature decoupling of representations. In a typical false belief scenario, the agent would not make a statement about her belief; enhanced P300 is, thus, attributed to the deviation of her belief from reality compared to a true belief condition. In a lying situation, the liar misrepresents her true belief when communicating it to an audience, and the P300 response is attributed to the deviation of her words from her true belief. The present findings are consistent with this explanation because given the basic design in which the story protagonist acquires a belief and makes a statement about it, the absence (Experiment 1) and presence of an audience (Experiment 2) focus the participants’ attention to the two types of representational discrepancy that, respectively, define false belief and lie situations in the literature. Hence, we conclude that the common involvement of P300 in the two domains indicates a sensitivity to representational discrepancy at a general level.

A related implication is that the sensitivity of P300 to representational discrepancy is situation‐dependent. Note that the deviation of words from belief in FS, Experiment 1, and the deviation of belief from reality in FB, Experiment 2 did not give rise to enhanced P300 compared to the other conditions. According to some previous studies, P300 differences reflect levels of endogenous attention allocation under different conditions (Volpe et al., 2007), especially when such conditions involve cues or stimuli that are considered by the observer to be relevant to himself or herself (Gray et al., 2004). In the FS condition in Experiment 2, the element of deception together with its emotional and moral overtones (Davis & Whalen, 2001; Grèzes et al., 2004) might stand out and mark the condition as the most attention catching and relevant under a communicative context, compared to the other two conditions. The importance of belief‐reality consistency was overshadowed. This contrasts with Experiment 1, which did not include a communication partner in the story; it was the consistency between belief and reality that stood out to define the situation.

### PEER REVIEW

The peer review history for this article is available at https://publons.com/publon/10.1002/brb3.3021.

## Supporting information

Supplement Material

## Data Availability

The data that support the findings of this study are available from the first author upon reasonable request.
