# Supplementary material for: P300 as a correlate of false beliefs and false statements
Source: Brain Behav. 2023 Apr 19;13(6):e3021. doi: 10.1002/brb3.3021 (PMC10275538; doi:10.1002/brb3.3021)
Supplement: Supplementary file 1 — Supplement Material [file BRB3-13-e3021-s001.docx]

Supplementary Materials

**Randomization Tests**

In the GFP analysis, the selections of the time windows and electrodes were based on the peak measurements of the brain responses; this procedure could be sensitive to random noises. To address possible bias due to the measurement procedure, non-parametric randomization tests equivalent to the original repeated-measures ANOVA, follow-up one-way ANOVA, and t-tests analysis were conducted. In these tests, the condition labels (FB, FS, TB) and electrode labels were shuffled within each participant. The peak measurement procedure described in the GFP analysis were repeated. Based on the GFP, the peak responses in the P300 / LSW search windows were identified and the 20 ms /200ms measurement window was applied to construct the P300 and LSW time windows. Electrodes showing the strongest responses in the P300 and LSW time window were identified, respectively. Brain response averaged among the peak electrode and it’s corresponding neighbouring electrodes were averaged for the following analysis. The mean sum of square values for the main effects and interaction effect in the repeated measures ANOVA and follow-up one-way ANOVA, and the differences in mean brain responses of the follow-up t-test were calculated. Then, the shuffling procedure was repeated 1000 times to generate null-effect sampling distributions for each of the statistical tests. The exact probabilities (p-values) of each test reported in the results were obtained by comparing the mean sum of square values and differences in mean brain responses of the original non-shuffled data with the corresponding null-effect sampling distributions.

**Experiment 1**

The results of the non-parametric randomization testes were same as the results of the original analysis. The ANOVA results showed a significant main effect of EIOI (*p*<.001), a significant main effect of condition (*p*<.001), and a significant interaction between EIOI and condition (*p*=.048). Follow-up one-way ANOVA was conducted on P300 and LSW, separately. P300 were significantly different among three conditions (p<.001), whereas LSW were not (p=.148). Post-hoc analyses revealed that FB elicited larger P300 than both TB, p<.001, and FS, p < .001. FS and TB did not differ in P300, p=.23.

**Experiment 2**

The same analysis protocol as Experiment 1 was conducted. The results of the non-parametric randomization testes were similar as the results of the original analysis. The main effect of condition was marginal significant (*p*<.001) while the main effect of EIOI was not significant (*p*=.934). The two-way interaction between EIOI and condition was significant (*p*<.001). Follow-up one-way ANOVA with condition as independent factor was conducted on P300 and LSW, separately. Both P300 (*p*<.004) and LSW (*p*<.001) were significantly different among three conditions. Post-hoc analyses revealed that FS elicited larger P300 than both FB, *p* < .001, and TB,  *p* = .009. FB and TB did not differ in P300,  *p* = .329. FB elicited larger LSW than both FS, *p* = .003, and TB, *p* < .001. FS elicited larger LSW than TB, *p* = .022.
